# Exploration of Point-of-Care Ultrasonography for Silicone Breast Implant Rupture Detection and Classification

**DOI:** 10.3390/medicina60020306

**Published:** 2024-02-10

**Authors:** Jae-Hong Kim, Yun-Gyoung Kim, Keun-Yeong Song, Hyung-Guhn Lim, Jeong-Pil Jeong, Jung-Youp Sung, Angela-Soeun Lee, Heung-Kyu Park

**Affiliations:** 1 THE W Clinic, Seoul 06038, Republic of Korea; stenka@hanmail.net; 2 Department of Surgery, Bundang Jesaeng General Hospital, Seongnam 13590, Republic of Korea; 3 Department of Breast Surgery, Gwangju Suwan Hospital, Gwangju 62247, Republic of Korea; 4 Department of Radiology, Gwangju Suwan Hospital, Gwangju 62247, Republic of Korea; 5 Samsungyubang Breast Clinic, Busan 48104, Republic of Korea; 6 BBC Plastic Surgery Clinic, Changwon 51209, Republic of Korea; 7 Korean Society of Breast Implant Research, Seoul 03186, Republic of Korea; 8 Department of Surgery, Breast Cancer Center, Gachon University Gil Medical Center, 21, Namdong-daero 774beon-gil, Namdong-gu, Incheon 21565, Republic of Korea

**Keywords:** breast implants, rupture, ultrasonography

## Abstract

*Background and Objectives*: The surge in breast-related surgeries in Korea underscores the critical need for an accurate early diagnosis of silicone breast implant-related issues. Complications such as BIA-ALCL and BIA-SCC add complexity to breast health concerns, necessitating vigilant monitoring. Despite advancements, discrepancies persist between ultrasonographic and pathologic classifications of silicone implant ruptures, highlighting a need for enhanced diagnostic tools. This study explores the reliability of ultrasonography in diagnosing silicone breast implant ruptures and determining the extent of silicone migration, specifically with a focus on guiding potential capsulectomy based on pathology. *Materials and Methods*: A comprehensive review of medical records encompassing 5557 breast implants across 2790 patients who underwent ultrasound-assisted examinations was conducted. Among the screened implants, 8.9% (249 cases) were diagnosed with silicone breast implant rupture through ultrasonography. Subsequently, 89 women underwent revisional surgery, involving capsulectomy. The pathological analysis of 111 periprosthetic capsules from these cases aimed to assess the extent of silicone migration, and the findings were juxtaposed with the existing ultrasonographic rupture classification. *Results*: The diagnostic agreement between preoperative sonography and postoperative findings reached 100% for silicone breast implant ruptures. All eighty prosthetic capsules exhibiting a snowstorm sign in ultrasonography demonstrated silicone migration to capsules upon pathologic findings. *Conclusions*: High-resolution ultrasonography emerged as a valuable and reliable imaging modality for diagnosing silicone breast implant ruptures, with a notable ability to ascertain the extent of free silicone migration to capsules. This diagnostic precision is pivotal in informing decisions about potential capsulectomy during revisional surgery. The study advocates for an update to the current binary ultrasonographic classification, suggesting a more nuanced categorization into three types (subcapsular, intracapsular, and extracapsular) based on pathology.

## 1. Introduction

In Korea, breast cancer has emerged as the predominant concern among women, leading to a surge in post-mastectomy breast reconstruction surgeries and elective silicone breast procedures for cosmetic enhancement. The growing prevalence of these interventions underscores the critical need for accurate early diagnosis, particularly in the case of silicone ruptures, a condition often devoid of apparent symptoms.

The implications of silicone migration gain added significance, notably during pregnancy and childbirth, carrying risks associated with silicone infiltrating the mammary glands. Untreated silicone ruptures pose substantial health risks, potentially interfering with cancer screening and increasing the likelihood of secondary damage, which raises concerns about undetected breast cancer and its potentially fatal consequences.

Notably, an emerging concern in recent years is the occurrence of breast implant-associated anaplastic large cell lymphoma (BIA-ALCL) and squamous cell cancer (BIA-SCC) after breast implant-based mammaplasty [1,2,3,4,5]. This newfound challenge adds complexity to the landscape of breast-related health issues, further emphasizing the need for vigilant monitoring and timely intervention. This emphasizes the necessity for vigilant monitoring and timely intervention, as highlighted by both the Korean Ministry of Food and the US Food and Drug Administration, stressing routine breast implant check-ups in response to the rising cases of silicone ruptures and the emerging threat of BIA-SCC [6].

Given these considerations, timely treatment remains imperative to mitigate potential fatal outcomes. This urgency highlights the pivotal role of accurate diagnostic tools. This study seeks to address this pressing need by investigating the reliability of high-resolution ultrasonography in diagnosing silicone breast implant ruptures. Additionally, we propose an updated classification system based on pathological findings, contributing to a more comprehensive understanding of breast health in the context of surgical interventions, encompassing both silicone implant-related complications.

In the realm of implant-based aesthetic and reconstructive surgery, the persistent challenges of rupture and capsular contracture continue to necessitate revisional procedures [7,8,9,10,11]. These complications, which stand as primary reasons for revisional surgery, emphasize the critical need for accurate diagnostic tools and classification systems. Currently, mammography and ultrasonography typically serve as the initial steps in the diagnostic workup, while magnetic resonance imaging (MRI) is considered the gold standard [12]. Several studies have compared the diagnostic accuracy of mammography, ultrasound, and MRI in detecting implant ruptures [13,14,15,16,17]. Recently, high-resolution ultrasonography (HRUS) has emerged as a valuable tool for diagnosing implant-related complications, including ruptures, and has demonstrated efficacy in identifying device types and manufacturers [9,18,19,20,21,22].

Despite these advancements, there remains a critical gap in research, particularly in understanding silicone migration to capsules through pathology. While accurately diagnosing ruptures in asymptomatic patients is crucial, determining the extent of silicone migration to capsules, adjacent tissues, or lymph nodes holds equal importance in planning reoperations [23]. The precise diagnosis of silicone migration is integral to the planning of revisional surgery, particularly when considering the possibility of capsulectomy.

As we delve into the diagnosis and classification of silicone breast implant ruptures, discrepancies are observed between the existing classifications in ultrasonography and pathologic silicone migration categories (subcapsular, intracapsular, and extracapsular). Mammography and MRI, lacking posterior acoustic shadowing, categorize silicone implant ruptures as intra- and extracapsular ruptures. In contrast, ultrasonography, which is capable of showing posterior acoustic shadowing (snowstorm signs), allows for a more nuanced classification. Ultrasonography reveals three distinct categories, addressing the need for a comprehensive diagnostic approach: (1) cases without posterior acoustic shadowing, (2) cases with posterior acoustic shadowing without silicone migration to adjacent tissues and regional lymph nodes, and (3) cases with posterior acoustic shadowing and silicone migration to adjacent tissues and regional lymph nodes.

To enhance our understanding of silicone implant ruptures, this study focuses on the precision achievable through ultrasound, hypothesizing three distinct cases. Our objectives are to rigorously verify these cases using ultrasound measurements and investigate their correlation with the existing pathology classification, uncovering their clinical significance and informing medical decision-making.

This study holds immense importance as its outcomes promise to unveil tools and methods for accurately detecting silicone implant ruptures and establishing a tailored pathology classification system. This contribution is pivotal in reducing necessary biopsies and minimizing secondary damage to patients, ultimately transforming the silicone implant rupture diagnosis landscape for a more precise, patient-centric, and clinically significant approach.

## 2. The Current Study

The primary focus of our study was to assess the feasibility of utilizing ultrasound for the detection of silicone breast implant rupture. Additionally, we aimed to determine the reliability of ultrasound in evaluating silicone migration to periprosthetic capsules, with the overarching goal of providing clarity in silicone rupture classification when compared with pathology.

To achieve these objectives, our study employed a comprehensive approach. We systematically investigated the accuracy of ultrasound in diagnosing silicone breast implant ruptures. Concurrently, we cross-matched the presence of silicone migration to the periprosthetic capsules using ultrasound and pathology assessments. The assessment of ultrasound’s ability to detect silicone breast implant rupture was conducted with a meticulous examination of imaging results. This involved evaluating the ability of ultrasound to detect subtle signs indicative of silicone leakage or rupture.

## 3. Method

This study involved the examination of medical documentation pertaining to 2790 women who underwent both ultrasound (US) examinations and surgery at THE W Clinic in Seoul, South Korea, over a span of five years (2017 to 2022). Approval for the study was obtained from the Internal Institutional Review Board of the Korea National Institute of Bioethics Policy (IRB No. P01-202301-01-028). The review board waived the requirement for informed consent, adhering to ethical standards outlined in the 1964 Declaration of Helsinki and its subsequent amendments. The Aplio i600 (Canon Medical System, Otawara, Tochigi, Japan) system with a 7–18-MHz linear transducer was used for ultrasonography.

A comprehensive review of medical records was conducted, encompassing 5557 breast implants in 2790 patients who underwent ultrasound-assisted examinations. The examinations were performed between 31 August 2017 and 31 December 2022 at a single center by a seasoned breast surgeon specializing in breast implant ultrasonography. The expertise of the surgeon spans 12 years, ensuring a consistent and high level of proficiency throughout the study. All ultrasound-assisted examinations adhered to a standardized routine breast implant checklist, specifically the Korean Society of Breast Implant Research checklist (KoSBIR checklist). This systematic approach aimed to ensure a thorough and consistent evaluation of breast implants during the ultrasonographic screenings.

### 3.1. Study Design

**Figure 1:**
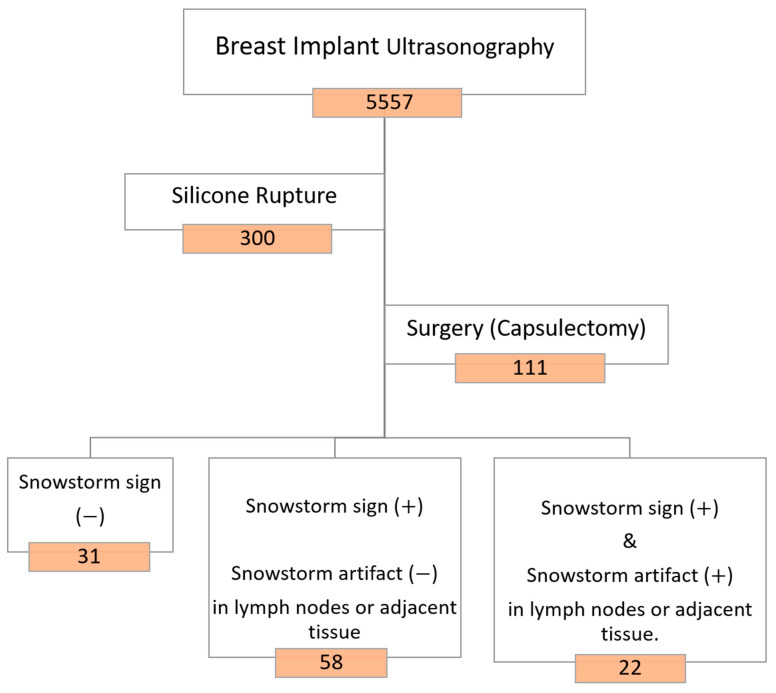
Study design (with intact implants).

The study population was categorically divided into two groups based on the condition of the silicone breast implants: patients with intact implants and patients with ruptured implants. For patients with identified silicone ruptures on ultrasound, further stratification was employed. These patients were classified into three groups based on the presence or absence of the snowstorm sign (posterior acoustic shadowing) and the occurrence of silicone invasion in the lymph nodes. A visual representation of this classification is provided in Figure 1. This stratification allows for a detailed analysis of the different manifestations of silicone breast implant ruptures, considering the distinctive ultrasonographic features observed during the examinations.

**Figure 2:**
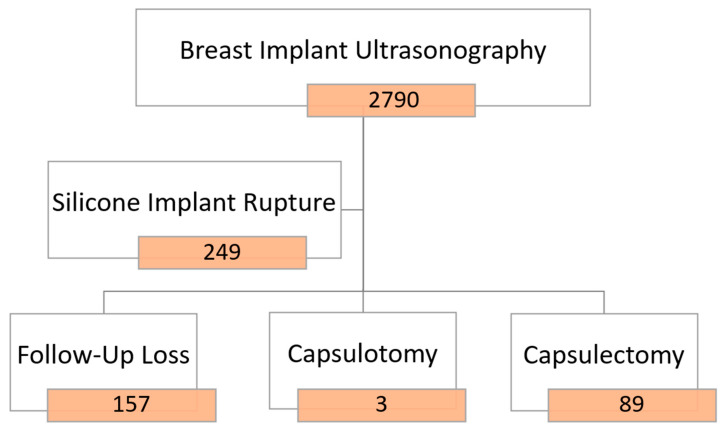
Study design (with ruptured implants).

Axillary lymph nodes were thoroughly assessed during the ultrasound examinations. Nodes were categorized as either normal or containing silicone, particularly focusing on the presence of snowstorm artifacts indicative of silicone infiltration. Among the 5557 breast implants (2790 patients) screened, images from 300 breast implants (249 patients) revealed evidence of silicone breast implant ruptures. Subsequently, 89 women (111 breasts) with confirmed silicone breast implant ruptures opted for surgery, specifically undergoing capsulectomy, as illustrated in Figure 2. Regrettably, 156 patients were lost to follow-up for revisional surgery at other medical sites.

One hundred and eleven periprosthetic capsules, extracted from 89 patients with ruptured implants, were thoroughly investigated for the presence and extent of leaked free silicone migration. The examination was conducted utilizing light microscopy (Olympus SZ61; Olympus Optical Co., Tokyo, Japan) coupled with a TUCSEN H series digital camera (Fuzhou Tucsen Photonics Co., Fuzhou, Fujian, China) at magnification rates of 40× and 100×.

### 3.2. Breast Implant Screening Ultrasonography, KoSBIR Checklist

All patients in this study were meticulously evaluated based on the Korean Society of Breast Implant Research (KoSBIR) breast implant ultrasonography checklist. This structured protocol serves as a comprehensive tool for the detailed description of breast implants following aesthetic or reconstructive implant-based mammaplasty. The utilization of the KoSBIR checklist ensured a structured and systematic evaluation of ultrasound images, allowing for the identification and documentation of various complications and specific characteristics associated with breast implants. Adopting the KoSBIR checklist standardized the evaluation process, contributing to enhanced consistency and reliability in data collection. This meticulously designed protocol is pivotal to the study’s methodological robustness, laying a strong foundation for investigating the accuracy of ultrasound in detecting silicone breast implant ruptures and associated complications.

The KoSBIR checklist comprises two essential components: a complication list and breast implant information extracted from the ultrasound images under investigation. The list of complications comprises instances of folding, with or without detachment, periprosthetic fluid accumulation, thickened capsules associated with capsular contracture, and rupture.

**Figure 3:**
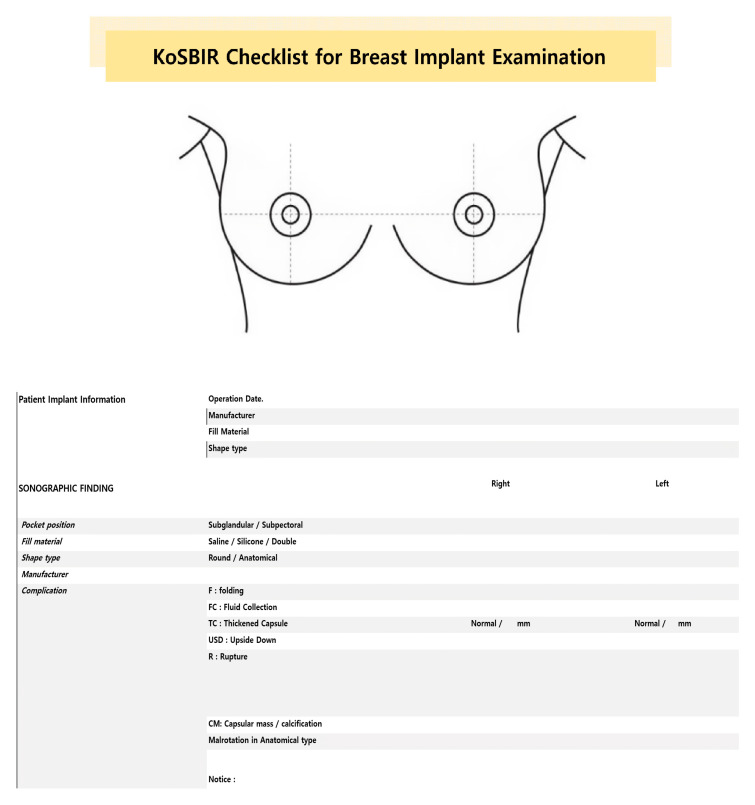
Checklist of Korean Society of Breast Implant Research.

In cases of rupture identified during ultrasound, a more in-depth evaluation is conducted to ascertain the presence of extracapsular silicone invasion into adjacent tissues and assess silicone invasion into lymph nodes. Additional complications considered encompass upside-down rotation and the presence of a capsular mass or calcification. The breast implant-related details encompass information about the pocket position, filler materials, shape, surface topography (including macrotexture, smoothness, and microtexture/nanotexture), and details about the manufacturer, as illustrated in Figure 3.

**Figure 4:**
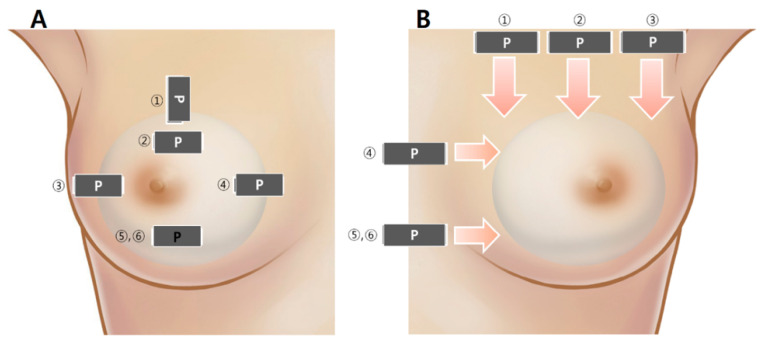
Position of breast implant ultrasonography. (**A**) Checkpoint for picture; (**B**) checkpoint for video (P is point of prove).

A minimum of six sonographic images and videos were captured for each breast, ensuring comprehensive information for a thorough assessment of individual breast implants, as illustrated in Figure 4. In Figure 4A, each number indicates the optimal location for specific information: (1) optimal position to check the breast implant pocket, (2) checkpoint for the upper part of the breast implant and periprosthetic space, (3) checkpoint for the lateral part of the breast implant and periprosthetic space, (4) checkpoint for the medial part of the breast implant and the periprosthetic space, (5) checkpoint for the inferior part of the breast implant and the periprosthetic space, and (6) optimal position for identifying the breast implant shell surface topography.

**Figure 5:**
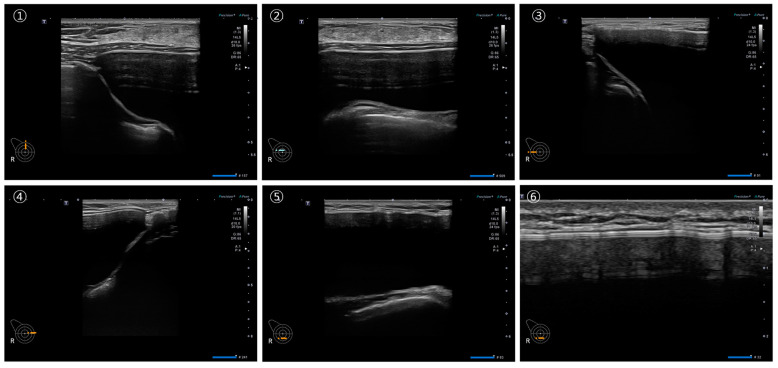
Examples of breast implant ultrasonography. ① US image of the lateral part of the breast implant and periprosthetic space, ② US image of the central part of the breast implant and the periprosthetic space, ③ US image of the medial part of the breast implant and the periprosthetic space, ④ US image of the upper part of the breast implant and the periprosthetic space, ⑤ US image of the lower part of the breast implant and the periprosthetic space, and ⑥ US image of the shell surface topography of the breast implant.

Figure 4B illustrates the optimal locations for specific video information, guiding the probe downward or horizontally toward the red arrow. Each number corresponds to assessing distinct aspects: (1) the lateral part of the breast implant and periprosthetic space, (2) the central part of the breast implant and the periprosthetic space, (3) the medial part of the breast implant and the periprosthetic space, (4) the upper part of the breast implant and the periprosthetic space, (5) the lower part of the breast implant and the periprosthetic space, and (6) the shell surface topography of the breast implant. Examples of ultrasonographic images strategically positioned for the optimal assessment of breast implant integrity are depicted in Figure 5.

### 3.3. Capsulectomy

**Figure 6:**
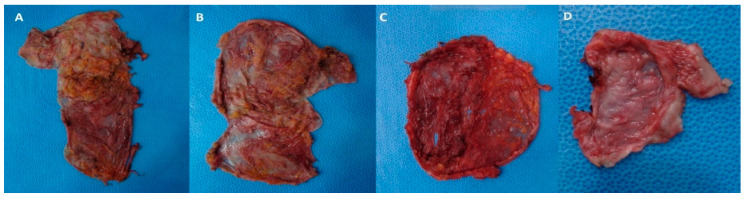
Types of capsulectomy. (**A**) Total capsulectomy, (**B**) near-total capsulectomy, (**C**) subtotal capsulectomy, and (**D**) partial capsulectomy.

Capsulectomy procedures were conducted through pre-existing incisions, including axillary, peri-areolar, and infra-mammary incisions. As depicted in Figure 6, four types of capsulectomy were employed: total capsulectomy (complete removal of the capsule), near-total capsulectomy (removal of over 90% of the capsule, complete removal of the capsule in the upper part, and almost complete removal of the capsule in the lower part), subtotal capsulectomy (removal of 70–90% of the capsule), or partial capsulectomy (removal of less than 70% of the entire capsule).

Following capsulectomy, a specific specimen was sampled and subjected to staining with hematoxylin and eosin dye. When ruptures displayed snowstorm signs in preoperative ultrasonography, a targeted capsule specimen was deliberately obtained based on preoperative design, aligning with the site that exhibited snowstorm signs in the preoperative ultrasonography.

### 3.4. Rupture Diagnosis

#### 3.4.1. Silicone Breast Implant (SBI) Rupture

**Figure 7:**
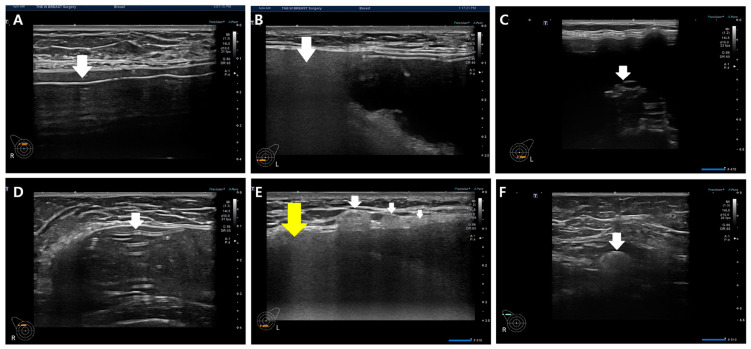
Various diagnostic clues in silicone breast implant rupture. (**A**) Free silicone (indicated by a white arrow), (**B**) snowstorm sign (indicated by a white arrow), (**C**) gel fracture sign (indicated by a white arrow), (**D**) stepladder sign (indicated by a white arrow), (**E**) adjacent silicone migration (indicated by a white arrow) with snowstorm sign (indicated by a yellow arrow), and (**F**) silicone migration to regional lymph nodes (indicated by a white arrow).

For the diagnosis of silicone breast implant (SBI) rupture, the presence of free silicone, characterized by spontaneous hypo-echogenicity in ultrasonography, or the identification of a shell tear manifesting as a disrupted thin linear hyper-echogenicity or gel fracture sign, was considered (Figure 7).

Gel bleeding is indicative of free silicone without shell tears. SBI rupture is characterized by a gel fracture and stepladder sign in implant integrity, as illustrated in Figure 7C and D. In some cases, rupture diagnosis may solely rely on the presence of gel fracture without the simultaneous occurrence of free silicone and shell tear in ultrasonography (Figure 7C). Upon confirming rupture, the evaluation and documentation of snowstorm signs and the extent of silicone migration to adjacent tissue or regional lymph nodes are performed (Figure 7E,F). This record is crucial information for physicians and patients, helping prevent unnecessary biopsies prompted by remnant silicone migration to adjacent tissues and regional lymph nodes.

#### 3.4.2. Current Classification of Silicone Breast Implant Rupture Scope

The existing silicone breast implant rupture classification distinguishes between intracapsular and extracapsular ruptures based on imaging diagnoses using mammography (MMG), ultrasound, or MRI. In the current framework, intracapsular ruptures are characterized by the absence of silicone migration to adjacent tissue in MMG, ultrasound, or MRI images. In contrast, extracapsular ruptures are identified by a snowstorm appearance in ultrasonography (Figure 7B,E) and the migration of silicone to adjacent tissue or regional lymph nodes, detectable by MMG and MRI. The classification by MMG and MRI into intra- and extracapsular ruptures stems from their inability to display posterior acoustic shadowing. In contrast, ultrasonography, with its ability to reveal posterior acoustic shadowing (snowstorm signs) and detect silicone migration to adjacent tissue or regional lymph nodes, introduces a more nuanced approach by dividing silicone breast implant ruptures into three types.

**Figure 8:**
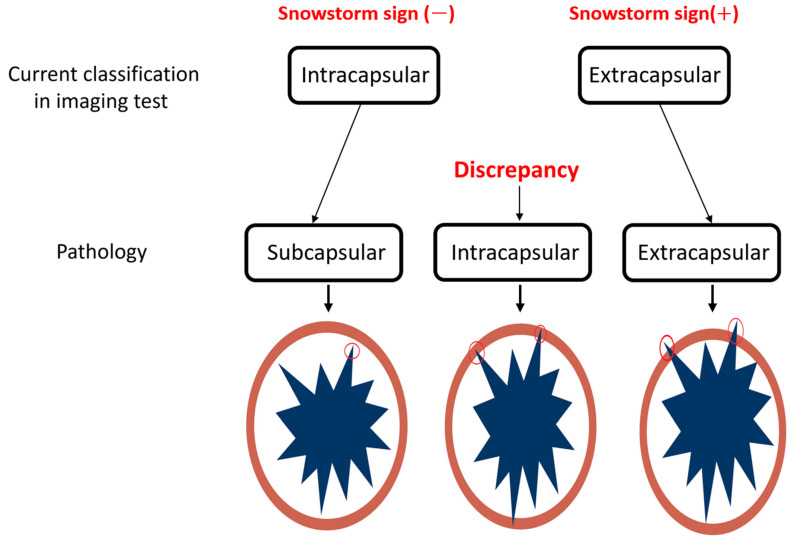
Current and pathologic division of silicone migration to capsules. (Red circle is a range of silicone migration).

This departure in ultrasonography from other imaging modalities forms the foundation for our study, where we aim to reconcile the discrepancies between the two ultrasonographic classifications and pathology. To address this disparity, we investigated 111 periprosthetic capsules in silicone breast implant ruptures by comparing snowstorm signs in preoperative ultrasound with silicone migrations to capsules identified through pathology. We cross-matched the two intra- and extracapsular rupture classifications with the three pathologic conditions of the capsules (Figure 8) in an effort to provide a more comprehensive and accurate understanding of silicone breast implant ruptures.

The pivotal aspect of this study revolves around the pathologic intracapsular silicone migration into the capsule, which serves as the linchpin for resolving the discrepancy between ultrasound (US) findings and pathology (Figure 7). When pathologic intracapsular rupture exhibits snowstorm signs in ultrasonography, the current classification categorizes it as an extracapsular rupture, deeming it a severe condition. Consequently, there is a need to explore the clinical significance of pathologic intracapsular rupture and compare it with pathologic extracapsular rupture. These distinct classifications hold divergent clinical implications for treatment and follow-up management, akin to the differentiation between intraductal and invasive carcinoma in breast cancer. Treatments for ductal carcinoma in situ and invasive ductal carcinoma significantly differ, emphasizing the importance of accurate classification.

Patients displaying snowstorm signs exclusively in ultrasonography, without silicone migration to adjacent tissue or lymph nodes, are diagnosed as intracapsular ruptures in mammography (MMG) and MRI. This diagnosis is due to the inability of MMG and MRI to showcase silicone embedding in capsules and the posterior acoustic shadowing (snowstorm sign). Numerous studies have reported a higher incidence rate of intracapsular rupture than extracapsular rupture. If the classification of silicone breast implant rupture is conducted using both ultrasonography and pathology, the prevalence of intra- and extracapsular ruptures may significantly vary. This highlights the need for a more nuanced and comprehensive approach to capture the diversity in silicone breast implant ruptures accurately.

## 4. Results

This study assessed the characteristics and concordance of silicone implant rupture diagnosis between preoperative ultrasonographic findings and postoperative gross findings in 89 patients. Table 1 provides an overview of the characteristics of these 89 patients who exhibited silicone breast implant rupture in preoperative ultrasonography.

The agreement in rupture diagnosis was 100% between preoperative ultrasonography and postoperative gross findings among the 89 patients. The majority of cases (88.8%) occurred within ten years. Unilateral rupture was more frequent, accounting for 75.3% of cases. Table 2 outlines the characteristics of the 111 ruptured breast implants. All cases involved aesthetic silicone breast implant-based mammaplasty (100%), conducted through a trans-axillary incision, and were asymptomatic in the majority of instances (88.3%). Round breast implants were predominantly used in these cases (84.7%).

Clinically, the macrotexture shell type implant accounted for 47.7%, while the smooth type constituted 46.8%. Ruptures were more frequent in round breast implants compared to anatomical types. Various manufacturers were identified, including Groupe Sebbin SAS, Boissy-l’Aillerie, France; HansBiomed Co. Ltd., Seoul, Korea; Allergan Inc., Irvine, CA, USA; Mentor Worldwide LLC, Santa Barbara, CA, USA; among others. Table 3 details the characteristics of 111 ruptured silicone breast implants observed in ultrasonography. Gel bleeding, indicative of free silicone without shell tears, was identified in two cases (1.8%). Most cases exhibited both free silicone and shell tears.

Table 4 presents the pathologic characteristics of 111 breast implants and capsules. Among the 111 ruptured implants, 80 (72.1%) were identified as extracapsular ruptures, displaying a snowstorm sign in preoperative ultrasonography. Silicone migration to capsules was examined using light microscopy in all 111 periprosthetic capsules, as detailed in Table 4.

Pathologic subcapsular rupture accounted for 31 cases (27.9%, Figure 7A). The most prevalent type was pathologic intracapsular rupture, observed in 58 cases (52.3%), where the snowstorm sign was present without silicone migration to adjacent tissue or regional lymph nodes in ultrasonography (Figure 7B). Pathologic extracapsular rupture, characterized by the snowstorm sign with silicone migration to adjacent tissue or regional lymph nodes in ultrasonography (Figure 7E,F), occurred in 22 cases (19.8%). Notably, silicone migration to regional lymph nodes was more frequent than adjacent tissue invasion in extracapsular rupture (Table 4).

**Figure 9:**
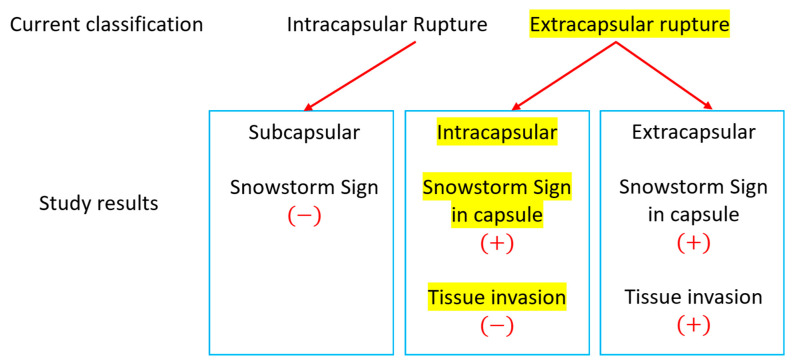
Correlation of silicone invasion with current classification and pathologic result by this study.

In a cross-match of all 111 capsules with ultrasonographic snowstorm sign presence, it was determined that all eighty capsules exhibited silicone embedding, inducing posterior acoustic shadowing (snowstorm signs) in ultrasonography (Figure 9). Figure 9 illustrates the study’s results, aimed at elucidating the discrepancy between the current two ultrasonographic findings and the three pathology classifications.

Therefore, pathologic intracapsular and extracapsular silicone migrations are identified as current extracapsular ruptures with positive snowstorm signs in the current definition. All fifty-eight cases of pathologic intracapsular silicone migration were matched with posterior acoustic shadowing due to silicone embedding in capsules in ultrasonography, resembling extracapsular ruptured cases.

**Figure 10:**
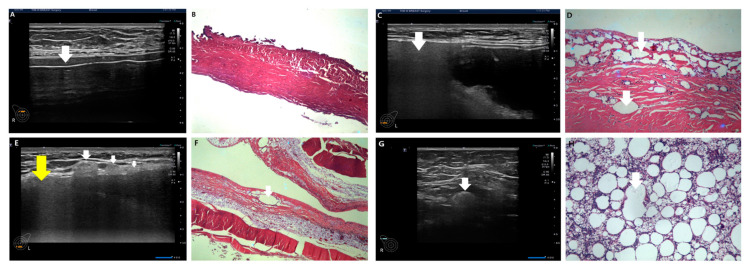
Ultrasonographic images of silicone breast implant rupture. Comparison between ultrasonographic findings regarding pathology. (**A**) Subcapsular silicone rupture in ultrasonography (a white arrow indicates free silicone); (**B**) pathologic finding of subcapsular rupture (there is no silicone migration in capsule); (**C**) intracapsular silicone rupture in ultrasonography (a white arrow indicates free silicone); (**D**) pathologic finding of intracapsular silicone invasion (a white arrow indicates silicone migration); (**E**) extracapsular silicone migration to adjacent tissue with snowstorm sign (snowstorm sign indicated by a yellow arrow, silicone migration to adjacent tissue is indicated by a white arrow); (**F**) pathologic finding of extracapsular silicone migration (a white arrow characterizes silicone migration to capsules); (**G**) silicone migration to regional lymph nodes (a white arrow indicates snowstorm artifact); and (**H**) pathologic finding of silicone migration to a regional lymph node(a white arrow indicates free silicone).

Pathologic subcapsular rupture (SCR) corresponds to the current intracapsular rupture, with no snowstorm signs and no silicone migration to adjacent tissue or lymph nodes in ultrasonography (Figure 10A,B). Pathologic intracapsular rupture (ICR) corresponds to the current extracapsular ruptures in which there is only a snowstorm sign (+) without silicone migration to adjacent tissue or lymph nodes (Figure 10C,D). Pathologic extracapsular rupture (ECR) corresponds to the current extracapsular ruptures in which there is a snowstorm sign (+) with silicone migration to adjacent tissue or lymph nodes (Figure 10E–H). Many silicone implant ruptures are considered intracapsular, although they show snowstorm signs in ultrasonography. Since they do not show silicone migration to regional lymph nodes or adjacent tissues, this misdiagnosis caused many silicone implant ruptures to be regarded as early stages (Figure 8C).

## 5. Discussion

Breast augmentation stands as the most prevalent cosmetic surgery, with around 300,000 females in the United States undergoing breast implant surgery for aesthetic and reconstructive purposes [24]. Recently, the explantation of breast implants has increased due to concerns related to BIA-ALCL (breast-implant associated anaplastic large cell lymphoma) and other implant-related illnesses [24,25]. Complications associated with breast implants encompass capsular contracture, malposition, rupture, rippling, seroma/hematoma, BIA-ALCL, and wound infection [26]. Moreover, rare cases of squamous cell cancer post-breast augmentation have been reported [4,5].

**Figure 11:**
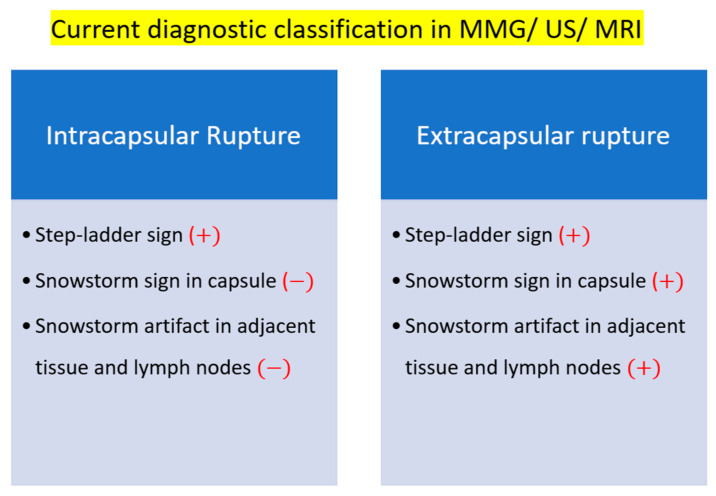
Current classification of silicone breast implant ruptures by various signs.

Common complications requiring reoperation in implant-based augmentation mammaplasty or post-mastectomy reconstruction include ruptures and a capsular contracture of Baker grade III/IV [27,28]. Diagnosing implant rupture proves challenging with only a physical examination [17,29,30]. Mammography faces limitations in diagnosing rupture, as approximately 80% of silicone breast implant ruptures lack extracapsular granuloma and lymph node silicone migration [7]. Breast implant ultrasonography, guided by FDA guidelines, has seen increased usage for the initial diagnosis of various implant-related complications [20]. The KoSBIR breast implant checklist provides a method for consistently obtaining breast implant information and potential complications [18,19]. Notably, all published studies have classified silicone breast implant ruptures as intracapsular or extracapsular without comprehensive comparison with pathology (Figure 11) [7,17,30].

Many studies previously reported a higher incidence of intracapsular rupture (80–90%) compared to extracapsular ruptures [17,31,32]. However, our current study, utilizing high-resolution ultrasonography, reveals that ruptures showing snowstorm signs were more prevalent, either with or without silicone migration to adjacent tissues or lymph nodes. Regional silicone migration to lymph nodes was more common than extracapsular silicone invasion into breast parenchyma in patients with extracapsular ruptures. The clinical impacts of pathologic intracapsular and extracapsular silicone migration differ, even though they exhibit the same snowstorm signs, emphasizing the need for capsulectomy in revisional surgery. The condition of silicone migration to capsules with snowstorm signs only holds the same clinical significance as the current intracapsular rupture (pathologic SCR), where there are no signs of silicone migration to extracapsular tissues. Immediate revisional surgery should be performed to prevent silicone migration to regional lymph nodes, which may transform into extracapsular rupture. A long-standing gel bleed could lead to free silicone migration to lymph nodes, indicating that gel bleeds should not be interpreted as intracapsular ruptures in the current classification system and may signify an initial rupture [32].

An annual breast implant examination is recommended for patients to prevent the exacerbation of silicone migration to regional lymph nodes or adjacent tissue, which could prompt non-radical silicone removal and unnecessary lymph node biopsies. While it has been reported that patients with current extracapsular ruptures may commonly present with symptoms [21], our study found that approximately 90% of patients with snowstorm signs in ultrasonography, with or without silicone invasion to adjacent tissue or lymph nodes, were asymptomatic. This suggests that extracapsular ruptures may be overlooked, underscoring the importance of regular follow-up for early diagnosis. Hölmich noted that there are rare cases where silicone might cause severe local problems in the event of silicone gel-filled breast implant rupture [33]. A regular ultrasound-assisted examination of the breast implant is crucial for early and accurate diagnosis in asymptomatic cases. Once a breast implant rupture is diagnosed, patients should undergo reoperation to remove the ruptured device and free silicone promptly. A delayed diagnosis of a rupture could result in the migration of free silicone to adjacent tissue and regional lymph nodes, making it challenging to perform a complete excision of the lesion [31]. Young women with silicone infiltration into the mammary glands may experience problems with lactation, and silicone lymphadenopathy could lead to the possibility of regional lymph node excision [18,23], potentially causing lymphedema.

## 6. Limitations

The present study does not entail a direct comparison between MRI and sonography. As ultrasonography is a real-time and operator-dependent examination, the results might not be readily generalizable. Furthermore, the study was carried out with a limited number of cases in a single center, and thus, a larger multicenter study would be essential to substantiate our findings. Notably, the loss of follow-up data from 156 patients seeking revisional surgery at other medical sites is a considerable limitation. This absence introduces a potential bias and challenges the study’s generalizability. As the outcomes of these cases remain unknown, the impact of their inclusion on the study’s overall findings is difficult to assess. Future research should address these limitations by employing strategies to enhance patient follow-up and engagement across multiple medical sites, ensuring a more comprehensive and representative analysis. The unavoidable challenge of maintaining consistent patient engagement underscores the need for comprehensive strategies in future research endeavors.

## 7. Conclusions

In summary, high-resolution ultrasonography emerged as a valuable and reliable imaging modality for diagnosing silicone breast implant ruptures. Its efficacy was particularly notable in discerning the extent of free silicone migration to capsules, facilitated by identifying posterior acoustic shadowing induced by silicone embedding. This capability is crucial for informed decision-making regarding capsulectomy in revisional surgery. To enhance the precision of silicone breast implant rupture classification based on pathology, ultrasonography advocates for a nuanced categorization into subcapsular rupture, intracapsular rupture, and extracapsular rupture. These findings underscore the pivotal role of ultrasonography in refining diagnostic accuracy and guiding appropriate interventions in the context of silicone breast implant ruptures.

## Figures and Tables

**Table 1:** Characteristics of 89 patients.

Variables	Values (%)
Age (years)	43.6 ± 10.8
Sex (male-to-female ratio)	0:89
Height (cm)	162.2 ± 4.6
Weight (kg)	53.9 ± 6.0
BMI (kg/m^2^)	20.5 ± 2.2
Day from previous surgery	4141.7 ± 2673.5
Less than 6 years	17 (19.1), 1278 ± 662
6~20 years	62 (69.7), 4081 ± 1198
More than 20 years	8 (9.0), 10,690 ± 2309
N/A	2 (2.2)
Rupture	
Unilateral	
Rt.	41 (46.1)
Lt.	26 (29.2)
Bilateral	22 (24.7)

**Table 2:** Characteristics of previous implant surgery in 89 patients.

Variables	Values (%)
Purpose of surgery	
Aesthetic	111 (100)
Reconstructive	0 (0)
Type of operation	107 (96.4)
Revision	4 (3.6)
Type of incision	
Trans-axillary	71 (64.0)
IMF	21 (18.9)
Peri-areolar	17 (15.3)
Other	2 (1.8)
Type of pocket	
Subpectoral	101 (91.0)
Subglandular	10 (9.0)
Shell type	
Macro-texture	53 (47.7)
Micro-texture	6 (5.5)
Smooth	52 (46.8)
Shape type	
Anatomical	17 (15.3)
Round	94 (84.7)
Manufacturer	
Sebbin	16 (14.4)
HansBiomed	4 (3.6)
Allergan	37 (33.3)
Mentor	23 (20.7)
Mcghan	3 (2.7)
Dow Corning	4 (3.6)
Silastic	3 (2.7)
Unidentified	21 (19.0)
Capsulectomy	
Partial	7 (6.3)
Sub-total	49 (44.2)
Near-total	29 (26.1)
Total	26 (23.4)
Symptom	
Yes	13 (11.7)
No	98 (88.3)

**Table 3:** Diagnostic findings in preoperative ultrasonography in 111 ruptured implants.

Variables	Values (%)
Free silicone only (Gel gel Bleedbleed)	2 (1.8)
Shell tear only	1 (0.9)
Free silicone with shell tear	108 (97.3)

**Table 4:** Divisions of silicone migration by pathology.

Silicone Invasion by Pathology	Values (%)
Sub-capsular	31(27.9)
Symptomatic	5 (16)
Asymptomatic	26 (84)
Intra-capsular	58 (52.3)
Symptomatic	7 (12)
Asymptomatic	49 (88)
Extra-capsular	
By symptom	22 (19.8)
Symptomatic	1 (4.5)
Asymptomatic	21 (95.5)
By site of silicone invasion	
Invasion of adjacent tissue	5 (22)
Invasion of regional lymph nodes	15 (69)
Invasion of both adjacent tissue and regional lymph nodes	2 (9)

## Data Availability

No data were created in this study.
